# Characteristics of Conventional and Microwave Sintered Iron Ore Preform

**DOI:** 10.3390/ma15072655

**Published:** 2022-04-04

**Authors:** Azhar Equbal, Mohammad Ali, Md. Asif Equbal, S. C. Srivastava, Zahid A. Khan, Md. Israr Equbal, Irfan Anjum Badruddin, Khalid Mohamed El-Hady, Sarfaraz Kamangar

**Affiliations:** 1 Department of Mechanical Engineering, Faculty of Engineering and Technology, Jamia Millia Islamia (A Central University), New Delhi 110025, India; zakhanusm@yahoo.com; 2 Department of Production Engineering, Birla Institute of Technology, Ranchi 835215, India; ali.cenation41@gmail.com (M.A.); sharadscs2@gmail.com (S.C.S.); 3 Department of Mechanical Engineering, Cambridge Institute of Technology, Ranchi 835103, India; equbal.asif@yahoo.com; 4 Mechanical Engineering Section, University Polytechnic, Aligarh Muslim University, Aligarh 202002, India; israr_equbal@yahoo.co.in; 5 Research Center for Advanced Materials Science (RCAMS), King Khalid University, Abha 61413, Saudi Arabia; sarfaraz.kamangar@gmail.com; 6 Mechanical Engineering Department, College of Engineering, King Khalid University, Abha 61413, Saudi Arabia; 7 Civil Engineering Department, College of Engineering, King Khalid University, Abha 61421, Saudi Arabia; kalhdi@kku.edu.sa

**Keywords:** conventional sintering, microwave sintering, preform, microhardness, cold crushing strength, hematite

## Abstract

In this study, compacted hematite (Fe_2_O_3_) preforms were made and sintered at various temperatures, such as 1250 °C and 1300 °C, using both conventional and microwave sintering methods. The density, porosity, microhardness, cold crushing strength, microphotographs, and X-ray diffraction (XRD) analysis of the sintered preforms were used to evaluate the performance of the two sintering methods. It was found that microwave sintered preforms possessed lesser porosity and higher density than conventionally sintered preforms owing to uniform heating of the powdered ore in microwave sintering method. Furthermore, it was also observed that microwave sintered preforms exhibited relatively higher cold crushing strength and hardness than conventionally sintered preforms. Thus, the overall results revealed that microwave sintering yielded better properties considered in the present study.

## 1. Introduction

**Figure 1:**
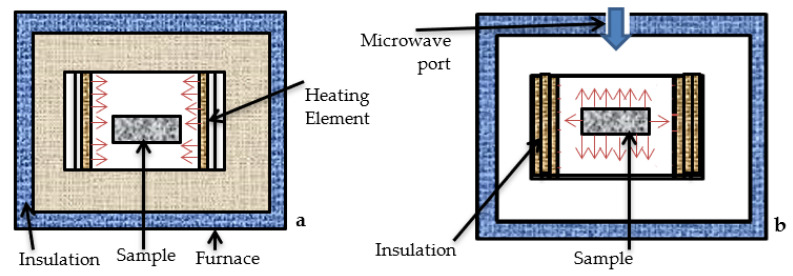
(**a**) Conventional sintering and (**b**) microwave sintering.

Sintering is the process of compacting a powder material to form a solid mass by applying a predetermined amount of heat or pressure without melting it to the liquid state [1,2,3]. Sintering is irreversible where the material is crushed, and the smaller particles release the surface energy to form bonds between them. Before sintering, the particles flow easily, but after sintering, the particles are bonded into a solid body to improve strength, density, and rigidity of the material [3]. Conventional sintering (CS) methods include solid state sintering, liquid phase sintering, and pressure assisted sintering. The details of these processes can be found elsewhere in the literature [4]. Long sintering time, high energy consumption, non-uniform heating, and poor temperature control are all disadvantages of the traditional sintering methods [4]. Microwave sintering (MWS) is a non-traditional sintering technology in which heat is generated inside rather than by surface radiative heat transfer from an external heat source [5]. RF (radio frequency) and MW (microwave) are used to generate heat at faster rate and evenly across the substrate in the MWS method. Figure 1a,b depicts the differences in the working principles of the CS (Figure 1a) and MWS (Figure 1b) methods, demonstrating that conventional sintering has a slower heating rate than microwave sintering. When compared to CS, microwave sintering offers the following advantages: (i) volumetric and uniform heating, (ii) faster sintering resulting in reduced sintering time, (iii) better temperature control, (iv) improved properties, and (v) minimum energy consumption [6,7]. A survey revealed that iron ore powder is sintered at a high temperature. Sintering is a pre-treatment process during iron making, and it is used to produce nodules of iron blended with small quantities of other minerals. Several studies on microwave sintering of powdered ore have been performed; however research on microwave sintering of iron ore is limited. Microwave sintering of selected iron ores has been examined. To expand the related research on microwave sintering of iron ore, we investigated the properties of microwave sintered hematite (Fe_2_O_3_). The results of the investigations were compared to those of conventional sintering done under comparable experimental settings to determine the advantages of hematite (Fe_2_O_3_) for MWS. The conventional sintering and microwave sintering procedures were used to produce sintered products of (Fe_2_O_3_) + limestone + coke + bentonite.

## 2. State of Art

This section outlines the previous work carried out on sintering of iron ore either through conventional and microwave sintering or comparison of both. In addition, the objective of the present research is listed at the end. Morteza and Omid [6] showed that microwave sintering provided better control than the traditional approach. They discovered that MWS achieved higher heat rates, and as a result, the sintering time was reduced in this method. They also indicated that MWS may be used to achieve greater thickness and grain appropriation. Anklekar et al. [8] used CS and MWS to investigate the sintered features of FC-0208 and FN-0208 organization steels. They reported that MWS delivered a larger sintered thickness, more prominent densification, and superior mechanical characteristics as compared to CS. The impact of heating mode on the sintering of Copper-SiC metal matrix composite was investigated by Ayyappadas et al. [9]. They found that MWS resulted in a significant densification as compared to regular sintering. When compared to traditional sintering, they found that faster handling in the microwave approach reduced processing time by 60%. Yang et al. [10] used microwave and conventional heating to produce SiC and SiC composites. They reported that at the same sintering temperature, microwave sintered SiC and SiC composites had superior flexural characteristics and toughness. Ramesh et al. [11] used MWS and CS techniques to investigate the mechanical properties and microstructural development of 3 mol percent yttria-settled zirconia sintering. They observed that MWS dramatically reduced the densification temperature, increased mass thickness, and improved mechanical properties. MWS was used by Yang et al. [12] to make Li_2_TiO_3_ burned rock. They compared microwave and traditional sintering of Li_2_TiO_3_ stones and discovered that MWS produced Li_2_TiO_3_ earthenware rocks with higher thickness, excellent mechanical properties, and a homogeneous microstructure. Chen et al. [13] also conducted a comparative study between MWS and CS of alumina grit. They suggested that the properties of microwave sintered alumina grit may be easily controlled by adjusting the sintering temperature and feed rate. They also found that microwave sintered parts possessed improved abrasive and mechanical capabilities, as well as a finer microstructure. Shukla et al. [14] investigated normal, microwave, and vacuum sintered parts and found that microwave sintering was the most vital and time-saving method. Padmavathi et al. [15] studied the effect of heating method and sintering temperature on the sintering of Al-1Mg-0.8Si-0.25Cu. They performed compactions under vacuum in conventional and microwave furnaces at various temperatures. They observed that microwave handling time was reduced by 58% as a result of greater warming rates and stronger densification. Further, they also noticed shrinkage when the sintering temperature was increased. The impact of factors on the MWS of BaTiO_3_ ceramics was investigated by Liu et al. [16]. They observed that MWS produced BaTiO_3_ earthenware in a considerably short drenching time. Furthermore, microstructure and dielectric constant analysis demonstrated that increasing the sintering temperature caused a significant shift in phase, with sintering at 1200 °C producing the best results. Brosnan et al. [17] used CS and MWS at 2.45 GHz to investigate the sintering energy and microstructural development of alumina tubes. To measure the temperature during MWS, they employed an infrared pyrometer aligned at 10 °C. They found that microwave-sintered parts reached 95% thickness at 1350 °C, while traditionally sintered parts attained the same thickness at 1600 °C. Li et al. [18] used CS and MWS methods to make SBN70 ceramics. They discovered that the MWS method produced high density earthenware in 2 h, but the CS procedure took 14 h. They also noticed that MWS of SBN70 pottery manufacture had better densification and grain uniformity. A similar study on the dielectric properties of Ba_0.95_Ca_0.05_Zr_0.25_Ti_0.75_O_3_ (BCZT) mass earthenware produced by conventional and MWS was performed by Mahajan et al. [19]. The effect of heating on density, quality, microstructure, and hardness of austenitic and ferritic tempered steel was investigated by Panda et al. [20]. They sintered the prepared compacts in a conventional furnace and a 2.45 GHz microwave furnace. They observed that in the microwave sintering method, 316L and 434L compacts reached the sintering temperature in a relatively short duration. They observed that MWS reduced the preparation time by about 90%. Chun et al. [21] studied the effect of microwave heating on the microstructures of iron ore pellets with coal during reduction. Optical microscopy and scanning electron microscopy were used by them to confirm the findings. They reported that microwave offered homogeneous heating and increased the growth and densification of metallic iron. The impact of heating mode on the characteristics and microstructure of the 92.5W-6.4Ni-1.1Fe compound was examined by Upadhyaya et al. [22]. They discovered that MWS reduced processing time by 75%, resulting in less coarsening of tungsten (W) grains leading to improved mechanical properties. The impact of warming mode on phase composition, microstructure, densification, and characteristics of iron and iron compounds was investigated by Annamalai et al. [23]. The findings of their research revealed that sintered compacts had equal densities independent of composition. They also discovered that all of the compacts shrank during sintering, resulting in greater densification, and that the mechanical qualities of compact sintered with microwave were superior to those of water atomized and wipe powders. An attempt to realize phase and microstructure control in Fe/Fe_2_SiO_4_-FeAl_2_O_4_ metal–ceramic by alternative microwave susceptors was performed by Gao et al. [24]. They proposed that use of microwave susceptors leads to effective control of the ratio of metallic and ceramic phases. Their results showed that the metal phase (Fe) and ceramic phase (Fe_2_SiO_4_, FeAl_2_O_4_) can be maintained however significant change in metal phase to ceramic phase was observed. The microstructures appeared as well-with the use of microwave susceptors. Literature manifests that microwave sintering of iron is relatively underexplored in the literature, with only selective ores of iron having been examined using the MWS process. As a result, the authors were inspired to expand their research on microwave sintering of iron ore, and hence, investigated the properties of microwave sintered hematite (Fe_2_O_3_). The results of the investigations were compared with those of conventional sintering done under comparable experimental settings to determine the advantages of hematite (Fe_2_O_3_) for MWS. The traditional sintering (furnace) and microwave sintering procedures were used to produce sintered products of iron ore (Fe_2_O_3_) + limestone + coke + bentonite in this study. The porosity, density, microhardness, cold crushing strength, microscopic examination, and X-ray diffraction (XRD) analysis of the sintered preforms were used to evaluate the sintering procedures’ performance.

## 3. Methodology

**Figure 2:**
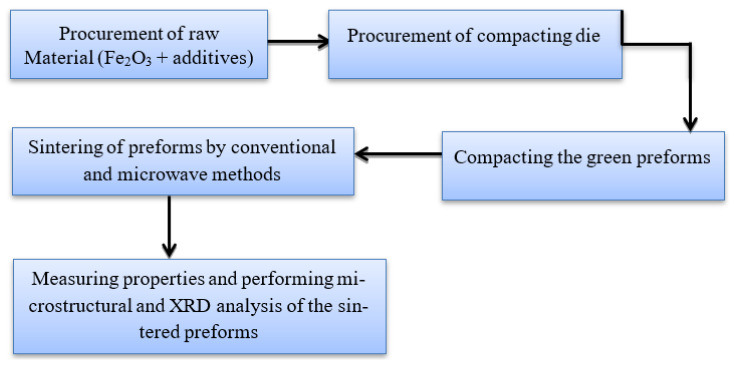
Workflow of investigation.

Based on the literature survey, Fe_2_O_3_ was selected as material for performing the experimental investigations. The approach used to conduct the current investigation is depicted in Figure 2. Despite of the fact that the hematite is inexpensive, readily available and abundant microwave sintering of this material is uncommon. Consequently, hematite along with additives was chosen for sintering.

**Figure 3:**
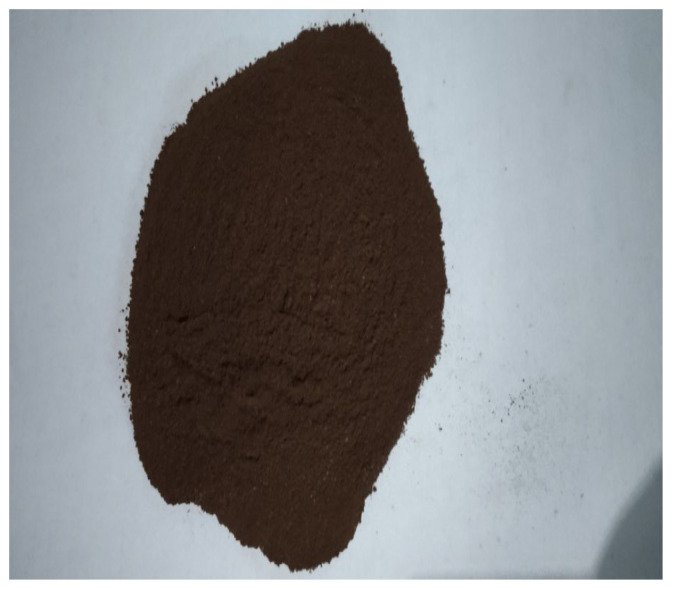
Powder form of hematite.

**Figure 4:**
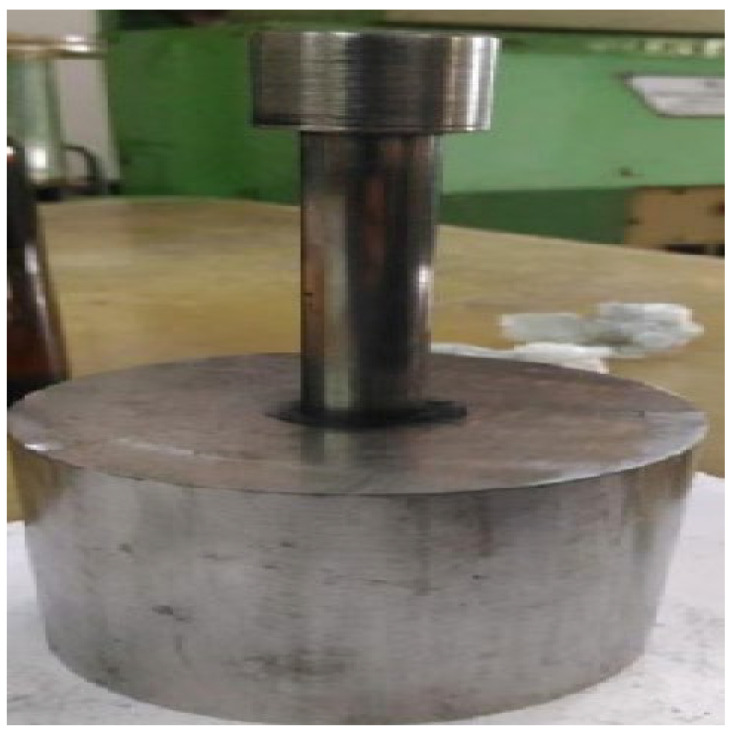
Compacting die.

**Figure 5:**
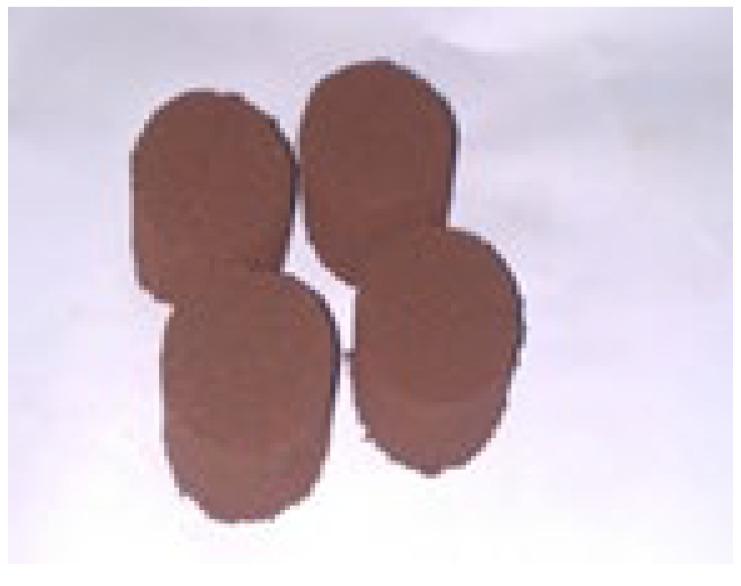
Preforms prepared after compaction.

The powdered form of hematite is depicted in Figure 3. To make the final powder, limestone (binding agent), coke, and moisture were added. The composition of the selected material is shown in Table 1. To ensure consistency in composition, the powder mixture was cup milled for 7–9 min at 1000 rpm using a vibrating cup mill (Insmart Systems, Telangana, India). Subsequently, 6% (wt. %) moisture was added to the powder and thoroughly mixed. The mixture was put into the die configuration (Figure 4) and compacted using a hydraulic compacting equipment to make compact preforms. The compaction method was used to improve the green density of the samples as well as to give correct shape to the powder. Compacted preforms were removed from the die for further processing. Fifteen cylindrical preforms with a diameter of 20 mm and a height of 9 mm were made. Figure 5 shows a few samples of the preform. Preforms were sintered using both conventional and microwave techniques at temperatures ranging from 1200 to 1300 °C. The preforms’ density, porosity, microhardness, and cold crushing strength were determined. The preforms were also subjected to microstructural examination and X-ray diffraction (Smart Lab, Rigaku, Japan) analysis.

### 3.1. Conventional Sintering of the Preforms

**Figure 6:**
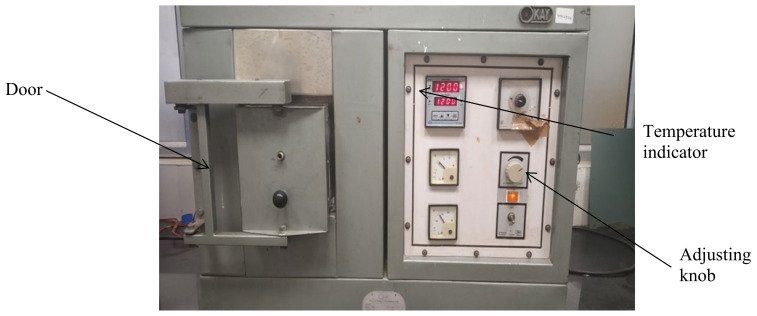
Electrical resistance based conventional heating setup.

**Figure 7:**
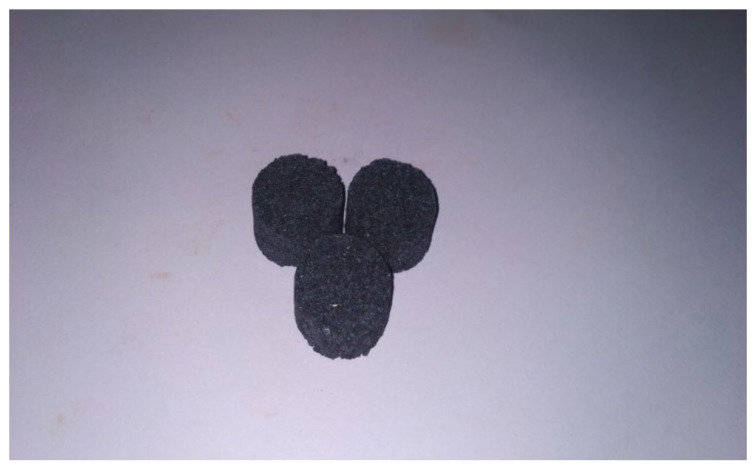
Preforms sintered in conventional furnace.

In this investigation, an electrical resistance-based traditional heating setup (OKAY Limited, Kolkata, India) was used. This arrangement included a furnace that could heat the material placed inside to around 1700 °C. Control switches for current and voltage regulation were available in the heating setup (Figure 6). Additionally, the temperature programmer of the setup might be used to retrieve temperature, heating rate, heating time, and other parameters. The preforms were placed on a ceramic crucible and introduced into a heating chamber, where they were heated at two different temperatures, i.e., 1250 and 1300 °C [25]. At these temperatures, the soaking time and the heating rate were set at 60 min and 5 °C/min, respectively. The heating rate was chosen based on the machine’s settings. The furnace was turned on after the preforms were placed inside, and the appropriate heating temperature was reached after around 4 h. Preforms were heated for approximately an hour at the desired temperature before the furnace was turned off. The preforms were cooled inside the chamber for 6–7 h before being removed [26]. As a result, normally sintered preforms were prepared, with a few of these samples depicted in Figure 7.

### 3.2. Microwave Sintering of the Preforms

**Figure 8:**
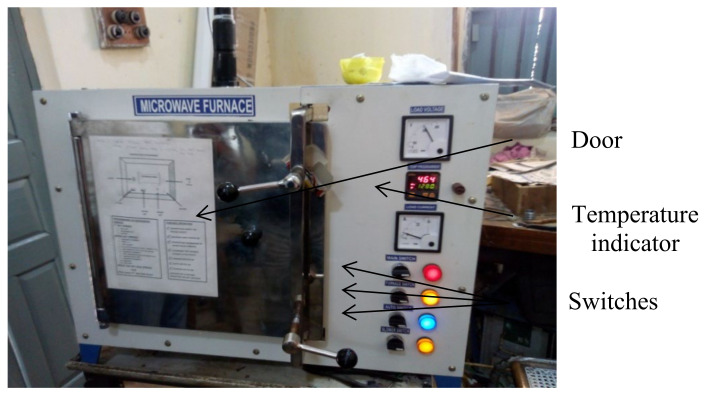
Microwave furnace setup.

**Figure 9:**
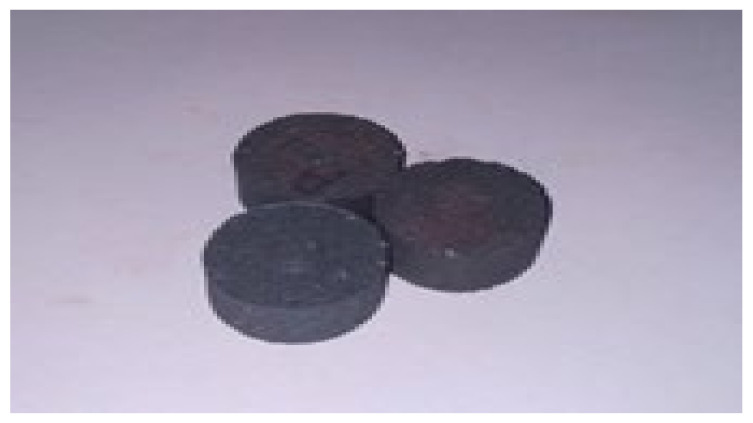
Preforms heated in microwave furnace.

The microwave furnace (VB Ceramics, Chennai, India) set up (200-KW) as shown in Figure 8 was operated at the frequency of 2.45 GHz (as per standards of ISM frequency). The main component of the furnace, i.e., the magnetron produced radio waves (microwaves) at the selected frequency with the help of both electric field and magnetic field (positioned in perpendicular directions). This setup had two magnetrons which produced the microwaves inside the chamber where preforms were kept for heating. The chamber was able to accommodate a specimen of size of 5×5×5 cm^3^. The preforms were heated using microwaves under vacuum created inside the chamber. An infrared sensor fixed at the top of the furnace inside the chamber was used to measure the heating temperature. Similar to conventional sintering, preforms were heated at 1250 and 1300 °C, respectively. The preforms were introduced into the chamber after being placed on the ceramic crucible. Because the cavity was small, only one preform could be heated at a time, and the remainders were heated separately under the same conditions. After inserting the preforms, the chamber was packed with the refractory bricks, and then, the furnace door was closed with metal foil wrapped from inside so that the vacuum could be maintained inside and no heat transfer could take place from outside. The program was manually fed into the setup with the predefined conditions through the control buttons provided on it. Preforms were cooled inside the furnace for about 5–6 h, and then, they were taken out. Microwave sintering was done at 1250 and 1300 °C, but soaking time and heating rate were kept at 30 min and 20 °C/min, respectively, so as to achieve the advantages of microwave sintering. The program was manually fed into the machine with the defined conditions through the control button provided on it. The conditions fed can be seen in the display screen provided. The heating temperature was measured by infrared sensor fixed at the top of the furnace inside the chamber. A few samples of the microwave sintered preforms thus prepared are shown in Figure 9.

## 4. Modules of Data Collection

The density and porosity of the preforms prepared using conventional and microwave methods were measured. Density is defined as the ratio of the unit mass of material to the unit volume of water. Porosity or void fraction is the measure of empty sites in a material, and it is expressed as a fraction of a volume. Bulk density ($\rho_{b}$) and porosity (*P*) of the specimens were determined using Equation (1) and Equation (2), respectively.

(1) $$\mathrm{Bulk}\;\mathrm{density},\;\rho_{b}=\frac{W_{D}}{W_{s}-S}\;\times \;\rho_{w}$$

(2) $$\mathrm{Apparent}\;\mathrm{porosity},\;P=\frac{W_{s}-W_{D}}{W_{s}-S}\;\times \;100$$

where $\rho_{b}$ is the bulk density in kg/m^3^, *W_D_* is the dry weight in kg, *W_S_* is the soaked weight in kg, *S* is the suspended weight in kg, and *ρ_w_* is the density of water in kg/m^3^.

The weight of the specimen measured on the electronic weighing machine under normal conditions is called dry weight. The weight of the specimen after it has been soaked for about an hour in distilled water and heated on a magnetic stirrer until the bubbles have stopped flowing out is called the soaked weight. The weight of a specimen suspended by thread and entirely immersed in water in a beaker is called suspended weight.

**Figure 10:**
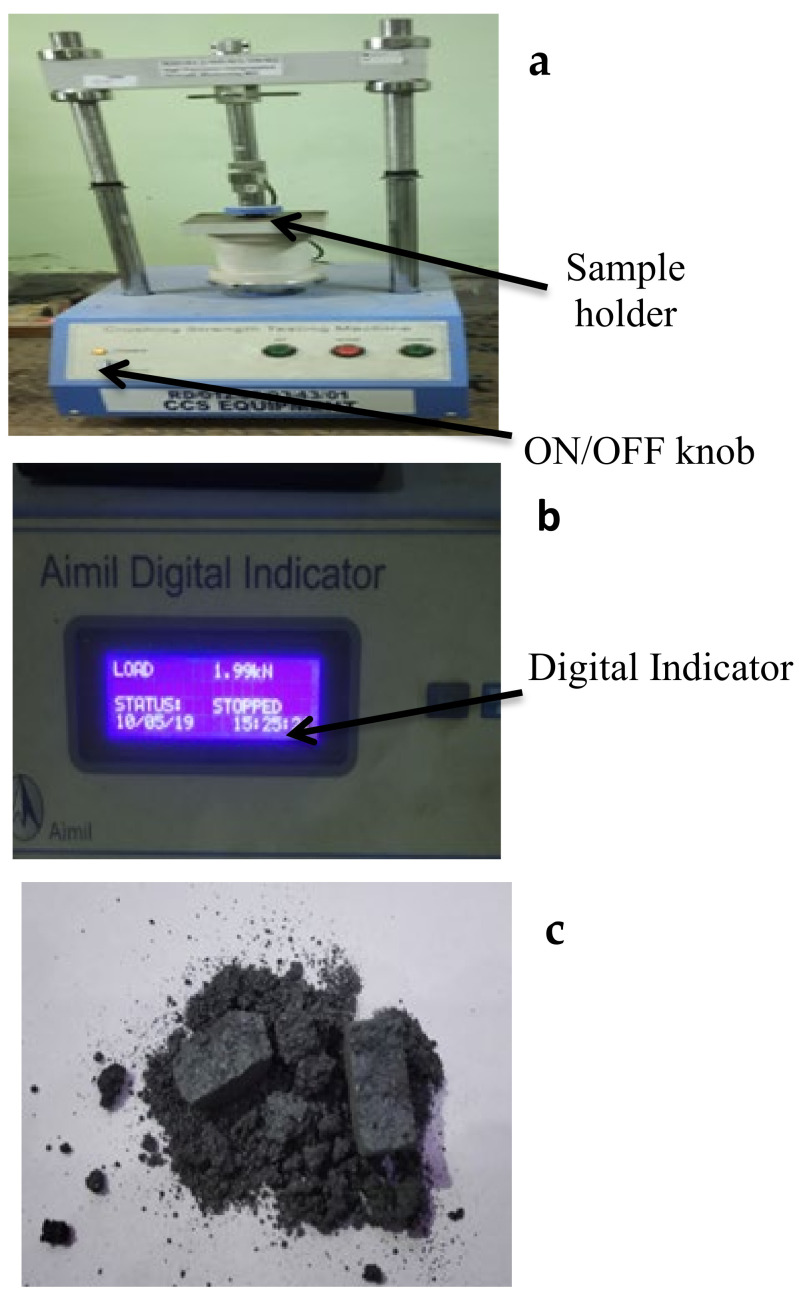
(**a**) CCS Tester, (**b**) digital indicator of CCS Tester, and (**c**) crushed sample after CCS test.

The determination of the cold crushing strength (CCS) of porous materials is also significant, and thus, a CCS tester (make: Aimil, India) was used to determine the CCS of the specimens, as shown in Figure 10a,b. The preform was rigidly attached to the CCS tester and subjected to a gradually increasing compressive load until it failed. Equation (3) was used to compute the CCS. Figure 10c shows a crushed sample following the testing.

(3) $$\mathrm{CCS}=\frac{F}{A}$$

where CCS is the cold crushing strength in kg/mm^2^, *F* is the force or load (in kg) at the point of failure and *A* is the initial cross sectional surface area in mm^2^.

**Figure 11:**
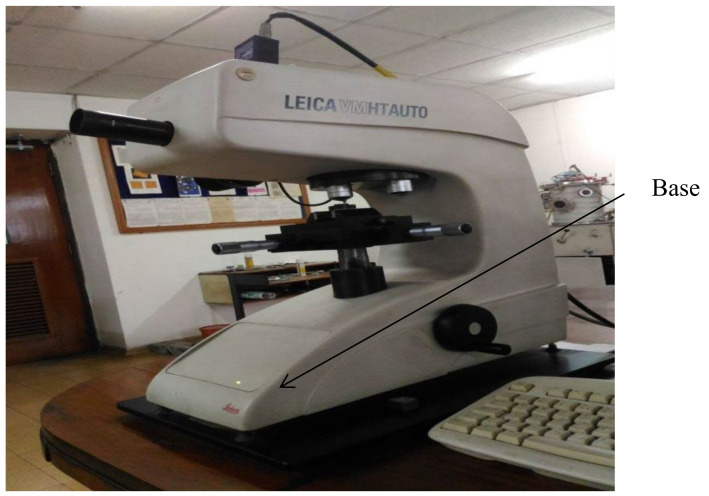
Microhardness tester.

**Figure 12:**
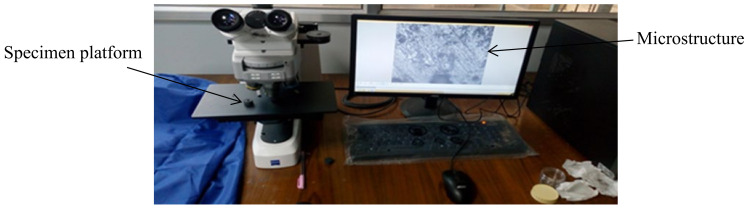
Optical microscope.

The microhardness of the samples was measured using microhardness tester of VHMT series (Figure 11) which was capable of providing Vickers hardness under a load varying from 1 g to 2 kg. The preforms were subjected to the fixed weights, and the necessary hardness was measured in HV. The microstructure of the sintered preforms was also determined using an optical microscope (Figure 12). Prepared preforms were polished using different grades of emery papers up to 2000 grade. The optical microscope was used to examine the preforms with the best densification. The crystalline structure of the produced preforms was also determined using an XRD (X-ray diffraction) test [27]. The wavelength of X-ray was diffracted by the crystals of the compound, and this property was widely used for studying crystalline structures. The samples for XRD analysis were 12 mm in diameter.

## 5. Results and Discussions

### 5.1. Density and Porosity

**Figure 13:**
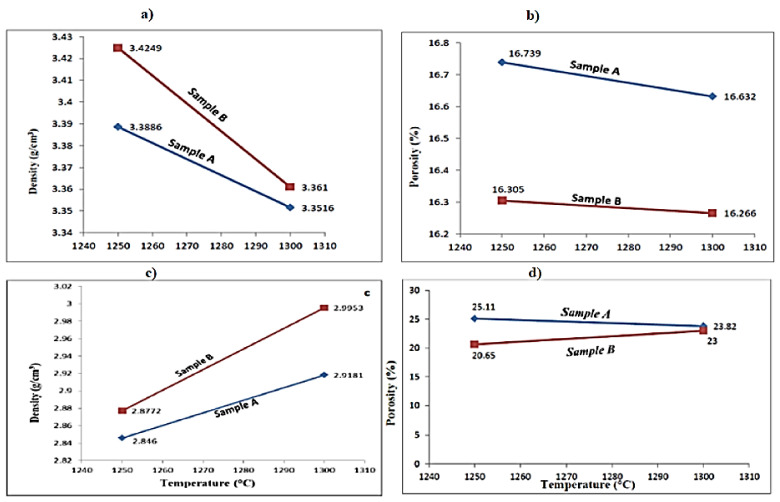
(**a**) Density and (**b**) porosity of microwave sintered samples; (**c**) density, and (**d**) porosity of conventionally sintered samples.

The density and porosity of the sintered preforms were computed using Equations (1) and (2), respectively. The various weights involved in the computation were taken in gram (g), and the volume was taken in cubic centimeters (cm^3^). For each of the sintering temperatures, 1250 and 1300 °C, two samples (Sample A and Sample B) were considered to observe variation in the properties due to processing conditions, and subsequently, a better sample was considered for further tests. Table 2 and Table 3 present the results of density and porosity measurements for the samples prepared through microwave sintering and conventional sintering, respectively. It is evident from Table 2 that preform sintered at 1300 °C is less porous than the one sintered at 1250 °C, which implies that porosity decreases with rise in sintering temperature due to the superior diffusion of iron ore (hematite) at increased temperature. It may be noted that the porosity of the sintered samples is a function of temperature, and therefore, no separate method was used to reduce porosity manually. Microwave sintered samples had less porosity than traditionally sintered samples at the same sintering temperature, as shown in Table 2 and Table 3. This is due to the fact that microwave sintering heats the samples from the inside out, allowing for improved iron ore diffusion. Furthermore, Table 2 and Table 3 show that the density of microwave sintered samples is higher than that of traditionally sintered samples, owing to the fact that improved diffusion in microwave sintered samples lowers porosity, resulting in a smaller space between molecules. A graphical representation for density and porosity measurements obtained using both sintering methods is presented in Figure 13a–d.

Figure 13a,b presents the results of density and porosity measurement for microwave sintered samples whereas Figure 13c,d shows the density and porosity measurements for samples sintered using conventional sintering. Figure 13a–d also reveals that microwave sintered samples had higher density and less porosity than conventionally sintered samples.

### 5.2. Cold Crushing Strength (CCS)

For the cold crushing strength test, preforms with higher densities were chosen from both microwave and conventional sintering methods. Sample B sintered at 1250 °C and sample B sintered at 1300 °C were taken from both sintering methods, based on the density measurement data shown in Table 2and Table 3. The results of the cold crushing strength test are shown in Table 4.

Table 4 clearly shows that the cold crushing strength or compressive strength of the microwave sintered preforms is much higher than the conventionally sintered preforms. Further, Table 4 also reveals that amongst all the considered samples, the cold crushing strength of sample B sintered at 1300 °C using microwave sintering method is the maximum. This is because of higher density and lesser porosity of this sample. Thus, it is inferred that high density and less porosity of the preform result in its higher cold compressive strength.

### 5.3. Microhardness Test

The Vickers hardness tester was used to determine the microhardness of sintered preforms manufactured using both methods. For the microhardness test, sample B sintered at 1250 °C and sample B sintered at 1300 °C with the higher density were chosen, similar to the cold crushing strength test. Individual preform microhardness values (in HV) were taken at four different loads: 50, 100, 200, and 300 gf, as shown in Table 5, which reveals that microhardness of the preforms increases with an increase in load and also with an increase in temperature. In general, a sample’s resisting capacity is self-adjustable and grows when more loads are applied; as a result, the microhardness of sintered samples increases as the load is increased. The microhardness of the preform improves as the temperature rises because it gets dry due to the removal of moisture, which aids in better fusion and so increases the preforms hardness and strength. Table 5 further shows that microwave sintered preforms have a better microhardness than conventional sintered preforms because they have less porosity and density, resulting in higher strength.

### 5.4. Optical Microscopy

**Figure 14:**
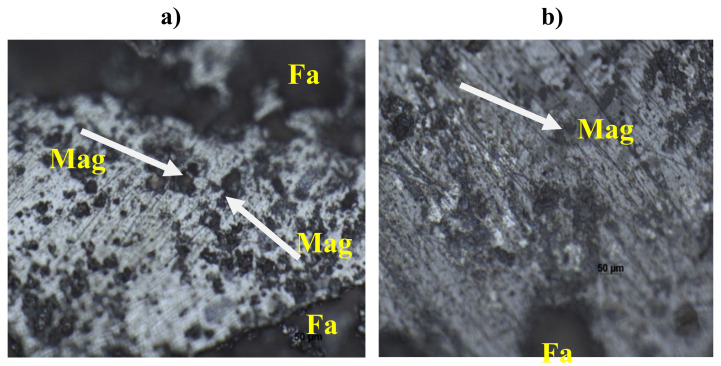
(**a**) Microscopic image of the conventionally sintered preform at 20X and (**b**) microscopic image of microwave sintered preform at 20X.

Figure 14a,b exhibits optical microscopic images of the conventionally and microwave sintered preforms, respectively, showing that hematite is the predominant phase present in abundance and that there is an enrichment of phases towards the center of the sintered preforms. Other phases, in addition to hematite, include Mag (magnetite) and Fa (Fayalite), as well as minor phases. The images also show that porosity reduces as the sintering process advances. The porosity of the microwave sintered preforms is likewise lower as can be seen in the images, indicating that this sintering method can produce higher hardness and strength [28]. The sample’s higher hematite content indicates that it has a superior oxidation capability after sintering, resulting in higher cold crushing strength. Less magnetite in the sample, on the other hand, indicates that the sintering process is less effective.

When comparing Figure 14a,b, it can be seen that microwave sintered preforms had more hematite and less magnetite than conventionally sintered preforms, implying that microwave sintering allows for better sintering [25]. The microscopic images also show that the microwave sintered preform has better features than that of the conventionally sintered preform.

### 5.5. XRD Analysis

**Figure 15:**
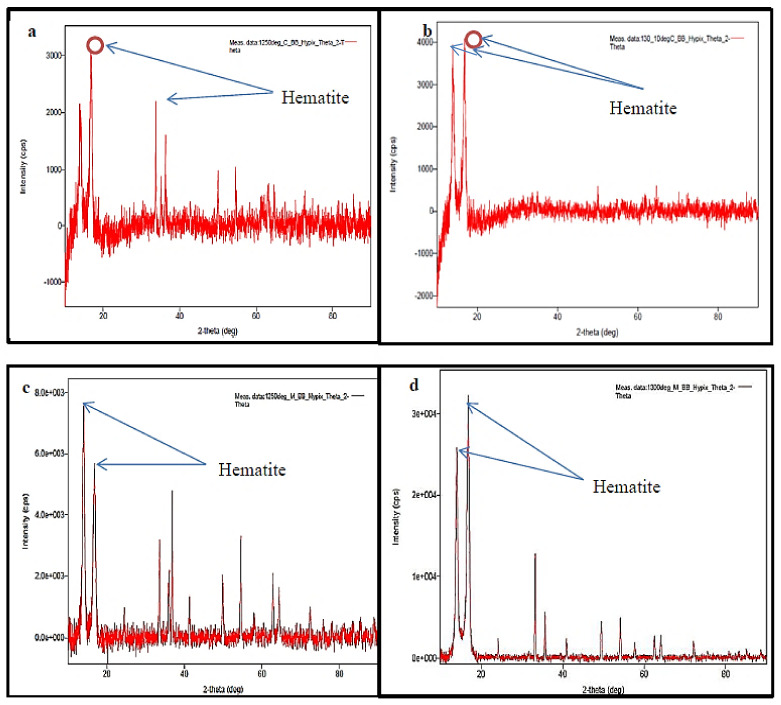
(**a**) Conventionally sintered at 1250 °C, (**b**) conventionally sintered at 1300 °C, (**c**) microwave sintered at 1250 °C, and (**d**) microwave sintered at 1300 °C.

The presence of iron in the sintered samples was detected using XRD analysis. Two conventionally sintered preforms were subjected to XRD analysis. In the first preform which was sintered at 1250 °C, two peaks of Fe_2_O_3_ (hematite) with high intensity of about 3200 and 2200 cps were found, as shown in Figure 15a, whereas in the second preform, which was sintered at 1300 °C, Fe_2_O_3_ was detected with the highest peak intensity of about 4000 cps and 4100 cps as exhibited by Figure 15b. For the two microwave sintered preforms, hematite was detected with a high intensity of 5900 and 7900 cps in the first preform which was sintered at 1250 °C as shown in Figure 15c while in the second preform which was sintered at 1300°C, peak with highest intensity was detected between 10 to 13 A° and 14 to 16 A° as depicted in Figure 15d. Microwave sintered preform showed the presence of iron at higher peak which implied that the hematite constituent was high and evenly distributed when compared with conventionally sintered preform.

## 6. Conclusions

Iron ore preforms were produced using a powder metallurgy technique and sintered at different temperatures (1250 and 1300 °C) using two sintering methods, namely, microwave sintering and conventional sintering, and their properties were characterized. It is observed that iron ore (Fe_2_O_3_) can be successfully sintered using both sintering methods. Experimental analyses were performed to measure and compare the densities, porosities, cold crushing strengths, and microhardness of samples prepared using both routes. In addition, XRD analysis was also carried out to show the presence of hematite in the finally prepared samples. Important conclusions drawn from the present research are the following:

- Microwave sintering is found to be a superior method since it takes less sintering time and produces sintered preforms with better microstructure.
- Microwave sintered preforms have less porosity and have a higher density than conventionally sintered preforms.
- Microwave sintered preforms have improved iron ore diffusion which provides more strength and micro hardness to them.
- Due to reduced porosity and higher density, microwave sintered preforms have relatively higher cold crushing strength.
- The microwave sintering method provides uniform heating of the powder material and also improves hematite distribution, as evident from the XRD analysis.

## Figures and Tables

**Table 1:** Composition of the material.

S. No.	Material	Composition
1	Iron ore (Fe_2_O_3_)	85%
2	Limestone	11%
3	Coke	4%

**Table 2:** Density and porosity measurement of microwave sintered samples.

Sl. No.	Samples and Sintering Temperature	Dry Weight (g)	Soaked Weight (g)	Suspended Weight (g)	Density (g/cm^3^)	Porosity (%)
1	A at 1250 °C	8.2733	8.6820	6.2405	3.3886	16.739
2	B at 1250 °C	8.2853	8.7732	6.3280	3.4249	16.305
3	A at 1300 °C	8.6331	9.0615	6.4857	3.3516	16.632
4	B at 1300 °C	8.5764	8.9915	6.4396	3.3610	16.266

**Table 3:** Density and porosity measurement of conventionally sintered samples.

Sl. No.	Samples and Sintering Temperature	Dry Weight (g)	Soaked Weight (g)	Suspended Weight (g)	Density (g/cm^3^)	Porosity (%)
1	A at 1250 °C	8.2108	8.9354	6.0504	2.8460	25.11
2	B at 1250 °C	8.3453	8.8799	6.0003	2.8772	20.65
3	A at 1300 °C	8.0988	8.7598	5.9844	2.9181	23.82
4	B at 1300 °C	8.1567	8.7975	6.0132	2.9953	23.00

**Table 4:** Results of cold crushing strength test.

Sl. No.	Samples	Load at the Point of Failure (kg)	Cross Sectional Area (cm^2^)	Crushing Strength (kg/cm^2^)
1	Conventional sintering, B (1250 °C)	202.920	3.1415	64.593
2	Conventional sintering, B (1300 °C)	227.396	3.1415	72.384
3	Microwave sintering, B (1250 °C)	498.641	3.1415	158.727
4	Microwave sintering, B (1300 °C)	530.250	3.1415	168.789

**Table 5:** Microhardness of the microwave and conventionally sintered samples.

Loads ►	50 gf	100 gf	200 gf	300 gf
Samples ▼				
Conventional sintering, B (1250 °C)	24.4 HV	30.4 HV	48.7 HV	70.5 HV
Conventional sintering, B (1300 °C)	29.8 HV	41.0 HV	53.1 HV	71.8 HV
Microwave sintering, B (1250 °C)	33.5 HV	43.8 HV	67.8 HV	91.0 HV
Microwave sintering, B (1300 °C)	33.8 HV	69.3 HV	75.5 HV	98.9 HV

## Data Availability

All data available in the manuscript.
